# Immunodeficiency with Autoimmunity: Beyond the Paradox

**DOI:** 10.3389/fimmu.2013.00077

**Published:** 2013-04-12

**Authors:** R. Bacchetta, L. D. Notarangelo

**Affiliations:** ^1^San Raffaele Telethon Institute for Gene Therapy (HSR-TIGET), Division of Regenerative Medicine Stem Cells and Gene Therapy, San Raffaele Scientific InstituteMilan, Italy; ^2^Division of Immunology and the Manton Center for Orphan Disease Research, Children's Hospital Boston, Harvard Medical SchoolBoston, MA, USA

The association of immunodeficiency and autoimmunity may represent a paradox, yet it has been described in an increasing number of conditions. Use of unbiased genomic approach to identify novel forms of primary immunodeficiencies (PIDs), along with in-depth functional studies in biological samples from affected individuals continue to unravel novel mechanisms underlying immune dysregulation in patients with altered ability of fighting pathogens. In particular, it has been clearly established that genetic defects that affect T and B cell development compromise not just the ability to generate a diversified repertoire of lymphocytes capable of recognizing multiple pathogens, but also impinge on mechanisms of central and peripheral tolerance, hence favoring autoimmune and inflammatory manifestations.

Yet, the diagnosis of autoimmune symptoms in the context of PIDs is troublesome, the prognosis unclear, and the treatment challenging. In the present collection of manuscripts, several experts in the field provide an overview of the spectrum of different forms of monogenic defects of the immune system manifesting also with autoimmunity, and discuss established and novel mechanisms involved in immune dysregulation.

Studies on patients with Immunedysregulation-Polyendocrinopathy Enteropathy-X-linked (IPEX) Syndrome, have paved the way to understand the phenotype arising from impaired peripheral tolerance due to dysfunctional regulatory T cells (Treg) expressing mutated FOXP3. However, this important T cell subpopulation can also be affected in other forms of PID, such as Wiskott–Aldrich syndrome (WAS) and adenosine deaminase (ADA) deficiency. In these disorders, the underlying genetic defect affects multiple cell types, resulting in impaired immune defense, but also poor Treg function. Similarly, *STAT5B* mutations disrupt an essential intracellular transcriptional activator for Treg cells, causing reduction of Treg number in affected individuals.

Autoimmune polyendocrinopathy-candidiasis-ectodermal dystrophy (APECED) is an autosomal recessive condition due to mutation of the Autoimmune regulator (*AIRE*) gene. Patients with APECED present with predominant organ specific autoimmunity and autoantibodies with multiple specificities. AIRE has been shown to play a critical role in allowing expression of self-antigens in the thymus, thereby permitting deletion of self-reactive T lymphocytes or their diversion to Treg cells. Thus APECED stands as the prototypic monogenic disorder of central T cell tolerance. While it is still questionable whether deficiency of AIRE also affects peripheral tolerance, recent data indicate that the autoimmune-associated tissue damage may not be primarily due to autoantibodies, but rather to autoreactive CD8^+^ T cells.

Moreover, recent studies in patients affected with Common Variable Immunodeficiency, a condition in which proper specific antibody production is deficient in favor of pathogenic autoantibody secretion, have highlighted the importance of mechanisms that control B cell development and receptor editing in maintaining immune homeostasis.

Finally, two manuscripts call the attention to the dual role of certain cell types and their ability to acquire different immunological functions depending on the environment in which they differentiate, as described for Th17 cells and dendritic cells, at the end of the Topic. Possibly, the future of medicine should aim to implement physiological plasticity and to empower epigenetics modifications in order to recover from inborn errors of Nature.

